# Beyond bias: A registered examination of the validity of using line bisection to measure non-lateralised attention

**DOI:** 10.1177/17470218241254761

**Published:** 2024-06-20

**Authors:** Alexandra G Mitchell, Aimal Ahmad Khan, Helen Stocks, Robert D McIntosh

**Affiliations:** 1 Center of Functionally Integrative Neuroscience, Department of Clinical Medicine, Aarhus University, Aarhus, Denmark; 2 Department of Psychology, The University of Edinburgh, Edinburgh, UK

**Keywords:** Attention, pseudoneglect, non-lateralised attention, line bisection

## Abstract

Line bisection is a task widely used to assess lateral asymmetries of attention, in which participants are asked to mark the midpoint of a horizontal line. The directional bisection error (DBE) from the objective midpoint of the line is the traditional measure of performance. However, an alternative method of studying the bisection behaviour, the endpoint weightings method, has been proposed. This method produces two measures of performance: endpoint weightings bias (EWB) and endpoint weightings sum (EWS). While EWB measures attentional asymmetry, it has been suggested that EWS quantifies the total (non-lateralised) attention allocated to the task. If EWS provides a valid index of non-lateralised attention, then changes in tonic and phasic arousal should systematically affect EWS. In this article, we formally tested this prediction, using time on task to manipulate tonic arousal and unpredictable auditory tones, presented simultaneously with line stimuli, to manipulate phasic arousal. Our registered analyses revealed that neither of our manipulations for tonic or phasic arousal significantly influenced EWS. Therefore, the null hypotheses cannot be rejected. An exploratory analysis of all trials and conditions revealed a significant reduction in EWS with time spent on task. However, the lack of any significant effect of the alerting tone on EWS suggests that EWS may not be a valid measure of generalised attention to the task.

## Introduction

### An alternative approach to the line-bisection task

The line-bisection task is a simple and common method for assessing lateral asymmetries of spatial attention. In the standard task, a horizontal line is presented to a participant, who is asked to mark its midpoint. The deviation of this response from the objective midpoint provides an index of attentional bias, directional bisection error (DBE), which is markedly rightwards in patients with left visual neglect following right hemisphere brain damage, indicating relative inattention to the left side (Schenkenberg et al., 1980). Neurotypical groups often show a subtle leftward DBE, suggesting relative *over-attention* to the left (“pseudoneglect,” see Jewell & McCourt, 2000). An alternative method for characterising the line-bisection behaviour, known as the “endpoint weightings” method, has also been developed (McIntosh et al., 2005). This method analyses the influence or “weighting” that each endpoint of the line has on the participant’s response, yielding two main summary indices of performance. The first is endpoint weightings bias (EWB), a measure of asymmetry that may be more sensitive than the traditional DBE to both neglect (McIntosh et al., 2005, 2017) and pseudoneglect (Mitchell et al., 2022). The second is endpoint weightings sum (EWS), which has been proposed as a measure of the total (non-lateralised) attention allocated to the task. This study aims to test whether EWS is a valid measure of non-lateralised attention for the line-bisection task.

The traditional line-bisection task takes DBE as its dependent variable, and the two key independent variables are the length of the line and its spatial position relative to the participant. Directional biases are amplified as line length increases, so left-sided-neglect patients make increasing rightward errors for longer lines (Bisiach et al., 1983; Butter et al., 1988; Nichelli et al., 1989), and healthy participants make increasing (though still small) leftward errors (see Jewell & McCourt, 2000). Patients with neglect generally show a negative (centripetal) spatial position effect so that bisection errors become relatively more rightward when the line is presented towards the patient’s left side and become relatively more leftward when the line is presented towards the right (Heilman & Valenstein, 1979; Nichelli et al., 1989; Schenkenberg et al., 1980). By contrast, neurotypical participants show a positive (centrifugal) spatial position effect, erring further leftward for lines in left space and further rightward for lines in right space (see Jewell & McCourt, 2000). These modulations mean that the magnitude and direction of DBE will differ depending on the length and spatial location of the presented lines.

The endpoint weightings method provides an alternative framework for administering and analysing the line-bisection task, which takes advantage of response modulations across different stimulus lines (McIntosh et al., 2005). The method begins by recasting the traditional task in terms of the dependent and independent variables involved. The new dependent variable is simply the horizontal position of the response relative to the midline of the workspace (or any other fixed reference), and the new independent variables are the positions of the left and right endpoints of the line, relative to the same fixed reference point. The task is administered as a series of lines, presented one at a time, in which left and right endpoints are varied independently. The endpoint weightings analysis then quantifies the degree to which the response position (P) is modulated by changes in the left (L) and right (R) endpoint positions. The left and right “endpoint weightings” are given by slope coefficients obtained by regressing P on L and R. A perfect bisection behaviour would produce symmetrical endpoint weightings of 0.5 because a shift of an endpoint causes the objective midpoint to shift by exactly half as much in the same direction. The endpoint weightings quantify the independent influences of left and right endpoints and predict how the responses will change when the endpoint positions are varied (for instance, when line length or spatial position is manipulated). The endpoint weightings method, therefore, gives an alternative mathematical description of familiar patterns of bisection behaviour.

### Line bisection may measure generalised attention as well as spatial bias

If we assume that an accurate bisection behaviour requires balanced spatial attention, then we may propose that an endpoint weighting indexes the amount of attention allocated to that end of the line (McIntosh, 2018; McIntosh et al., 2005). Given this interpretation, a participant’s performance may be summarised by two composite variables. The first is an index of lateralised attentional bias, EWB, given by the difference between the two endpoint weightings (right endpoint weighting minus left endpoint weighting), where positive values reflect a dominant influence of the right endpoint, and negative values a dominant influence of the left. Patients with left neglect have large positive values of EWB, and this index is more sensitive to neglect than the traditional index of DBE (McIntosh, 2018; McIntosh et al., 2005, 2017). Neurotypical participants have a slight negative EWB on average, and this index is similarly more sensitive to pseudoneglect than is DBE (McIntosh et al., 2005, 2019, Experiment 2). The second composite variable is the sum of the two endpoint weightings (EWS), which theoretically indexes the total amount of attention allocated to both ends of the line. Patients with neglect tend to have sub-normal values of EWS (i.e., EWS < 1; McIntosh, 2018; McIntosh et al., 2005, 2017), and neglect is well-known to be associated with reduced overall attentional resources (see Robertson, 1993). The endpoint weightings analysis may, therefore, be capable of measuring non-lateralised attentional allocation, as well as lateralised attentional bias, for the line-bisection task. The present experiment aims to test the validity of EWS as a measure of non-lateralised attention in a neurotypical sample.

If EWS does index non-lateralised attention, then it should be modifiable by manipulations to tonic (sustained) and phasic (transient) arousal, where tonic arousal refers to general wakefulness and engagement, and phasic arousal refers to a short term readiness to respond, for instance, following a warning signal (Sturm & Willmes, 2001). One established way to manipulate tonic arousal is via testing time, with prolonged performance of a task reducing arousal due to fatigue and/or boredom (Benwell et al., 2013; Dufour et al., 2007; Manly et al., 2005). One way to manipulate phasic arousal is via a salient event such as an auditory tone, which may act as a warning signal or can be an uninformative accessory stimulus causing a brief surge in physiological arousal (Hackley & Valle-Inclán, 1999; Jepma et al., 2009; Nakano, 2002; Robertson et al., 1998). In the present experiment, we manipulate tonic and phasic arousal to test the hypothesis that EWS is a valid measure of non-lateralised attention for the line-bisection task. Our methods and expected effect sizes are informed by prior published and unpublished studies, as described below.

### Predicted effects of time on task (tonic arousal)

**Figure 1.**
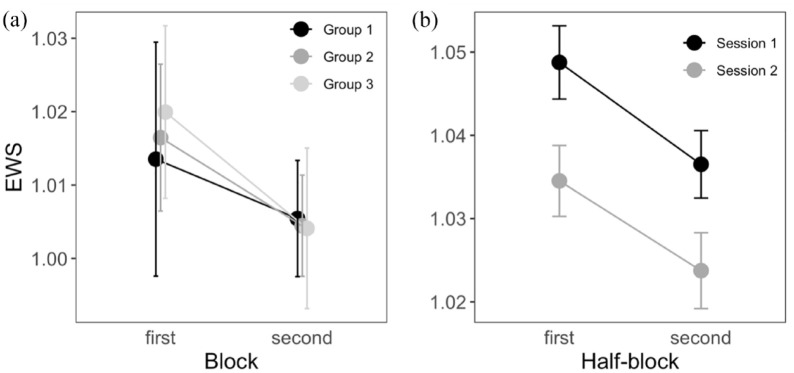
Reduced EWS over testing blocks in two prior studies. (a) EWS was reduced in the second line-bisection block relative to the first, after intervening prism (or sham) adaptation, in three groups of 12 participants (replotted from McIntosh et al., 2019, Experiment 2). (b) EWS is reduced both within sessions (between first and second half-blocks) and between testing sessions 9 days apart, in 215 participants (replotted from Mitchell et al., 2022).

The endpoint weightings method has been applied mainly to the study of paper-and-pencil line bisection by patients with spatial neglect (McIntosh, 2018; McIntosh et al., 2005, 2017). However, one study of neurotypical adults used an endpoint weightings analysis for a computerised (mouse-controlled) line-bisection task (McIntosh et al., 2019, Experiment 2). This experiment involved two extended blocks of line bisection (90 trials), which served as pre- and post-tests for an intervening (10 min) period of prism adaptation. Two groups of participants were adapted to leftward-displacing prisms, with a third, control group exposed to neutral lenses (*n* = 12 per group). No significant effect of prism adaptation was observed for measures of bisection asymmetry (EWB or DBE) or for EWS. However, a significant main effect of block was found, such that EWS was slightly reduced in the post-test relative to the pre-test across all groups (total *n* = 36). The reduction in EWS was small (mean 0.01, CIs −0.02 to −0.005) but statistically robust (Cohen’s *d* = −0.57, Figure 1a). This change was interpreted as a general reduction in attention due to declining (tonic) arousal over the course of the session.

Recently, we ran a large online study, in which 215 adult participants performed two blocks of computerised line bisection (80 trials), spaced 9 days apart (Mitchell et al., 2022). In an exploratory analysis of the effect of time on EWS, we separated each block into first and second half-blocks (40 trials each; Figure 1b). We observed a consistent reduction in EWS between the first and second half-blocks (mean −0.012, CIs −0.015 to −0.008; Cohen’s *d* = −0.44), consistent with declining tonic arousal due to fatigue. There was also a reduction in EWS between the first and second sessions (mean −0.014, CIs −0.019 to −0.008; Cohen’s *d* = −0.33). This effect of session cannot be so easily explained by fatigue, but it is possible that, because the task was already familiar, the participants were less motivated or less engaged in the second session and were allocating less attention to it. EWS may thus be sensitive to any factor that affects the level of general attention to the task, whether fatigue- or motivation-related. Therefore, time on task is used to manipulate tonic arousal, and it is worth considering that this may involve fatigue and/or other motivational factors.

These two studies showed patterns consistent with an effect of tonic arousal on EWS (Figure 1), supporting the hypothesis that this is a valid index of non-lateralised attention. However, these findings were exploratory, and neither experiment was designed to test this prediction. In the present study, we will conduct a confirmatory test of the effect of tonic arousal on EWS, using a time-on-task manipulation. Based on our prior data, we expect an effect size for a tonic arousal manipulation of *d* > 0.4 (McIntosh et al., 2019). However, to account for a possible overestimation of this effect size, due to selectively following up prior significant effects, we will target a conservative minimum effect size of *d* = 0.3.

Although we expect EWS to reduce with time on task in the present experiment, close consideration of Figure 1 suggests that this result could be ambiguous. Neurotypical participants generally have a mean EWS that is higher than one, so a slight reduction in EWS with time on task can move its value closer towards one. As already noted, perfect bisection performance would have symmetrical endpoint weightings of 0.5; therefore, as the sum of the two endpoint weightings, the theoretical optimum value for EWS is one, although EWS is often slightly greater than one (Figure 1). If EWS reduces across blocks, yet also gets closer to one, this could either reflect reduced non-lateralised attention due to declining tonic arousal or enhanced performance due to more task practice. Both theories can explain the reduction in EWS across sessions in Figure 1b. However, while the effect of time-on-task cold be ambiguous, it is possible to disambiguate attention and performance effects by including manipulation of phasic arousal. As EWS can range above one, it is possible that manipulations increasing arousal to the task can increase EWS above baseline levels. If phasic arousal is increased, the attention hypothesis predicts an increase of EWS, while the performance-enhancement hypothesis would predict that EWS should decrease to become closer to one. This critical manipulation of phasic arousal is described below.

### Predicted effects of phasic alerting

**Figure 2.**
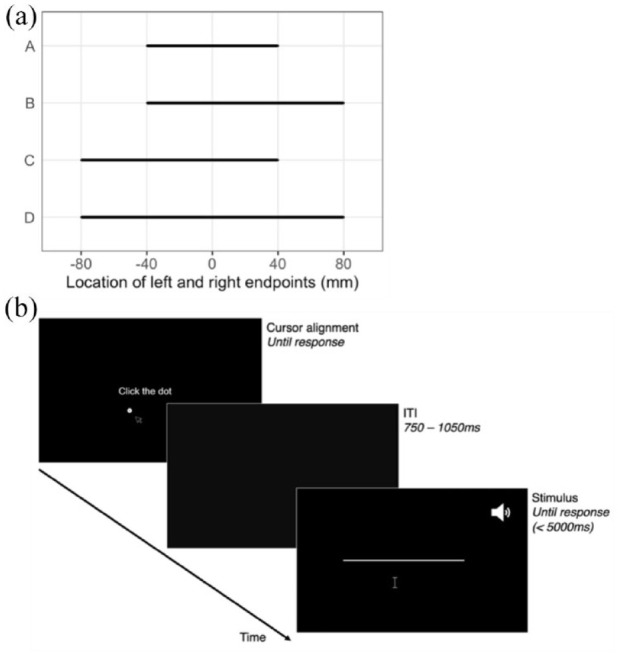
Methods. (a) The four-line stimuli, as defined by the location of their end left and right endpoints from the screen centre (0); during the experiment, lines are presented individually in white on a black background. (b) An example trial sequence. On half of the trials, a (short or long) tone is played simultaneously with the onset of the line stimulus.

We have previously used auditory tones to test the effect of phasic alerting on EWS in 33 healthy adult participants performing a computerised line-bisection task on a touchscreen with a stylus (Ahmad Khan, 2020). The bisection stimuli were configured for an endpoint weightings analysis, by factorially crossing two left endpoint positions (L = −40 mm and −80 mm) with two right endpoint positions (*R* = 40 mm and 80 mm, see Figure 2a). Participants performed 128 bisection trials in two blocks of 64 trials, with half of the bisection stimuli accompanied by a brief (300 msec) alerting tone (400 Hz or 1000 Hz), and the other half of trials in a silent baseline condition. Bisection responses were unspeeded, but more than 90% of responses were made within 3 s from the onset of the line. EWS was slightly but reliably higher in the tone condition, with a mean difference from baseline of 0.008 (CIs 0.0001 to 0.0167) and a standardised effect size of *d* = 0.36. The present study will include a confirmatory test of this effect of phasic alerting on EWS, using a similar tone manipulation, targeting a conservative minimum expected effect size of *d* = 0.3.

### Predicted effects of tonic and phasic alerting on lateralised attention

Our experiment focuses on the validation of EWS as a measure of non-lateralised attention for line bisection. However, studies have found generalised arousal to affect directional bisection bias which become relatively more rightward (i.e., reduction or reversal of pseudoneglect) with reduced tonic arousal due to time on task (Benwell et al., 2013; Dufour et al., 2007; Manly et al., 2005) or time since sleep (Manly et al., 2005). Similarly, increasing phasic arousal, via the use of unpredictable auditory tones, has been reported to reduce the rightward bias of neglect patients in a temporal order judgement task (Robertson et al., 1998). These effects are believed to arise because of a preferential involvement of the right hemisphere in general attentional arousal (Sturm & Willmes, 2001), so that higher arousal is associated with increased allocation of attention towards the (contralateral) left side, and vice-versa. However, we have not observed any lateralised effects of tonic arousal in previously cited experiments (McIntosh et al., 2019; Mitchell et al., 2022). Therefore, the present study provides an opportunity to re-examine this issue and test the effects of tonic and phasic arousal on the endpoint weightings measure of bisection bias (EWB). In further exploratory analyses, we will also include the traditional measure of DBE, for comparison with EWB. As it is not easy to obtain directly relevant effect size estimates from prior reports (e.g., Dufour et al., 2007; Manly et al., 2005) and the planned analysis of lateralised bias (EWB) is strictly secondary to our focus on non-lateralised attention (EWS), we powered our experiment for the primary analyses of EWS and assume equivalent statistical sensitivity for our secondary analyses of EWB.

## Preliminary manipulation check

An important preliminary step is to check that we can achieve an adequate manipulation of phasic alerting online, as the previous manipulations were lab-based. We therefore conducted a manipulation check, to confirm that our auditory tone is sufficient to induce phasic alerting effects in a GO-NOGO reaction time (RT) task (cf. Hackley & Valle-Inclán, 1999; Jepma et al., 2009; Nakano, 2002).

### Methods

#### Participants

Thirty-two participants were recruited online through Prolific (16 female and 16 male). All participants reported normal or corrected-to-normal vision and no prior history of neurological damage or underlying neurological conditions. Participants had to verify their identity on Prolific and have been active on the site within the 90 days prior to their participation. The study was limited to participants using laptops or desktop computers with a Windows Operating System. Ethical approval was obtained from the University of Edinburgh Psychology Research Ethics Committee (284/2021-3).

#### Quality checks and exclusions

One participant was removed prior to analysis due to incomplete data.

Two steps were used to obtain information about participant’s screen dimensions. First, in a standard online calibration step, participants pressed the left and right arrow keys to adjust the length of a horizontal line to match the width of a credit/debit card. The pixel-to-mm ratio derived from this step was used to scale the stimuli. Second, we used a custom calibration step where dots were presented sequentially at varying locations,^1^ and participants were instructed to click the location of each dot in turn. The coordinates of the response were recorded for each dot location, and a linear regression was fit to relate response coordinates with dot locations. If the model fit (r^2^) was <.90, then the participant was deemed not to be clicking on the dots with sufficient accuracy and was excluded from the analysis. Two participants were excluded by this criterion.

A final check was run to ensure that the audio was switched on for the experiment. A block of six “GO” and four “STOP” trials, presented in a random order, was completed. On each trial, an audio file of a male voice saying “GO or STOP” was presented, and participants were required to press the spacebar if they heard “GO” and to do nothing if they heard “STOP.” A feedback screen (“correct” or “incorrect”) appeared after the button press, or after 1,000 ms if no button was pressed. Participants were excluded from the analysis if they got fewer than four “GO” trials correct or fewer than two “STOP” trials correct. Eleven participants were removed by this criterion.

After these exclusions, total participant number was 18 (8 female, mean age = 22.7, *SD* = 3.2).

#### Procedure

The manipulation check consisted of a GO/NO-GO task, in which participants were instructed to press the spacebar as soon as they saw a circle on the screen. Before each trial, a screen appeared that instructed participants to press “ENTER.” After 750-1,050 ms, the two shapes appeared to the left and right of screen centre until the spacebar was pressed, or for a maximum of 1,000 ms. Shape combinations were either square and triangle; circle and triangle; or circle and circle. A circle was present on 60% of trials (GO trials), with no circle presented on the remaining 40% of trials (NOGO trials). Each trial was followed by a 500-ms feedback screen (“correct” or “incorrect”).

On 50% of GO and NOGO trials, a 500-Hz tone was presented simultaneously with the onset of the shapes. To prevent habituation, two tone types (long and short) were presented in equal numbers. The long tone had a duration of 40 ms with a rise/fall time of 60 ms, and the short tone had a duration of 78 ms with a rise/fall time of 3 ms; both tones had physical energy equivalent to that of an 80-ms tone with an instantaneous rise/fall (Dallos & Olsen, 1964). Participants were instructed to ignore the tones. After 20 practice trials, participants completed two experimental blocks, each consisting of 100 trials (30 GO-silent, 30 GO-tone, 40 NOGO-silent, 40 NOGO-tone).

### Results and conclusion

Participants performed at close to ceiling-level accuracy in all conditions (median accuracy ⩾ 98%). For each participant, the median correct RT was calculated for GO trials with and without a tone. RT was shorter for GO-tone trials (*M* = 452.1 ms, *SD* = 119.2) than for GO-silent trials (*M* = 461.5 ms, *SD* = 118.0). A one-sample *t*-test found that the paired RT difference (GO-silent − GO-tone) was significantly greater than zero (mean difference = 9.4 ms, *SD* = 16.16, t_17_ = 2.48, *p* = .023, Cohen’s *d* = −.58).

This manipulation check confirms that a task-irrelevant tone facilitates correct responses during the GO trials, with no obvious detriment to accuracy. These results are consistent with previous findings showing speeded response time with phasic alerting from auditory tones (Hackley & Valle-Inclán, 1999; Kiesel & Miller, 2007; Tudge & Schubert, 2016). This finding provides confidence that our tones are sufficient to induce a phasic alerting effect in an online context, showing that it is likely to be an adequate stimulus to test the sensitivity of EWS to phasic alerting in a line-bisection task.

## Main experiment: line bisection

### Methods

#### Participants

Our target sample size for this experiment was *n* = 119. Participants were recruited via Prolific, where they must have verified their identity and have been active on the site in the last 90 days. Recruitment was restricted to participants between the ages of 18 and 60 years, reporting normal or corrected-to-normal vision and no history of traumatic brain injury. The upper age limit was chosen to reduce the likelihood of hearing problems or undiagnosed cognitive impairment. We recruited a balanced split of male and female participants, although this was not essential to our study aims. As the study requires a keyboard and cursor, only participants using laptops or desktop computers were recruited. Participants who failed the audio check or who do not complete the entire experiment were not reimbursed for their participation, and their data were deleted. Participants who failed post hoc screen calibration checks were also removed from the study (see the *Quality checks* section).

#### Power considerations

The planned sample size of 119 was intended to provide .90 power to detect the targeted minimum effect size of *d* = .30, with a two-tailed significance criterion of *p* < .05. This targeted effect size is a conservative estimate of the expected effect size based on preliminary evidence outlined in the “Predicted effects of phasic alerting” section. Our final sample size of 110 (see the *Recruitment, quality checks and exclusions* section) had .90 power to detect a slightly larger minimum effect size than originally planned (*d* = 0.31).

#### Procedure

Before the main task, participants completed 10 questions of the Edinburgh Handedness Inventory (Oldfield, 1971), to provide descriptive information of the handedness characteristics of the sample, using short-form calculation of the laterality quotient (LQ), based on four of the sub-items (writing, throwing, toothbrush, spoon; Veale, 2014)

##### Quality checks

The audio quality check was adapted in accordance with attention check criteria set by Prolific^2^ and was conducted prior to the main experiment. Prior to the audio check, participants were advised to conduct the task in a quiet room with minimal distraction. First, a volume calibration step was conducted, where participants heard an example stimulus tone (500 Hz for 80 ms) and were instructed to adjust the volume of the tone (using the “up” and “down” arrow keys) until they could hear it clearly. This example tone was set to 70% of the total amplitude of both the audio check and stimulus tone, to ensure that participants hear the stimulus tones clearly throughout the experiment. Once completed, a voice speaking “one,” “two,” or “three” was played twice, and the participant was required to listen and then to indicate which number they heard by pressing one of three keys, “1,” “2,” or “3.” A block consisted of six trials (two trials per digit, randomised). If at least four responses were correct, then the participant was redirected to the screen measurement checks. If fewer than four responses were correct, participants were given the chance to repeat the audio check. If fewer than four responses were correct for the second attempt, participants could not continue with the experiment.

After the audio check, participants completed two further quality checks to obtain screen dimensions. Both these steps are outlined in the “Quality checks and exclusions” section above.

A final audio check was conducted at the end of the line-bisection task to help determine whether the volume remained on throughout the experiment. An audio recording of a voice saying the number “seven” was repeated twice, after which participants used a keyboard to enter the number they heard. Participants that did not enter the correct number were excluded from further analysis, as it is likely their volume was too low throughout the experiment or that they switched it off at some point during the experiment.

Finally, data from participants who did not complete the task correctly, or whose calibration and line-bisection fits were deemed invalid, were also removed from data analysis, as described in the “Data analysis and hypotheses” section.

##### Line-bisection task

The task was programmed in OpenSesame (Mathôt et al., 2012) and conducted online through the MindProbe JATOS server. To reduce the likelihood of participants changing their audio during the task, an instruction screen was presented at the beginning of the task, asking participants not to change the volume settings until the experiment was complete.

On each trial, 2-mm-thick white horizontal lines were presented individually on a black background at the vertical midline of the screen. Line stimuli were created using the combination of two left line endpoint locations (−40 mm and −80 mm) and two right endpoint locations (40 mm and 80 mm) relative to screen centre, producing four different line stimuli (Figure 2a). Participants used the mouse cursor to click on the perceived midpoint of each line. Prior to the beginning of each trial, participants were required to click on a single dot that appeared 25 mm below the middle of the screen. Dots appeared either 15 mm to the left or right of the midline, counterbalanced across the four different line stimuli. This step was included to ensure that the cursor position was moved away from the centre of the screen before each bisection judgement. Next, a blank screen appeared for 750–1,050 ms (jittered across trials), after which one of the four-line stimuli (Figure 2a) was presented until the response, or for a maximum of 5,000 ms (Figure 2b). If no response was made within 5,000 ms, a “too slow” feedback screen appeared, and that trial was excluded. On half of the trials, a tone was presented simultaneously with line onset, while the other half of the trials were silent. The two (short and long) 500-Hz tones used in the manipulation check were presented equally often for each line (see the “Procedure” section). To ensure that the participant clicked within the boundaries of the line, trials where the mouse click was not within the horizontal bounds or within 10 mm of the vertical bounds of the line were repeated at the end of the block.

Participants completed two experimental blocks (an early block and a late block), each consisting of 96 trials, with each line type presented 24 times per block (12 silent, 12 tone). Short and long tones were counterbalanced across line types, and trial order was shuffled randomly within blocks. The first experimental block was preceded by a practice block of 20 trials. Finally, an information screen was presented between blocks to give the participant a chance to take a short break, and participants were instructed to not take a break longer than 5 minutes.

### Data analysis and hypotheses

Only participants who passed the audio check took part in the main experiment, and only participants completing the full experiment proceeded to the analysis stage. Participants with complete data were excluded only if the fit between their responses and the calibration dot locations during screen calibration was less than r^2^ = .90 for either the x-axis or y-axis (see both *Quality checks and exclusions* and *Quality checks* section).

To maximise the likelihood that the bisection response was made while phasic alerting from a tone was still effective, trials were excluded if the response latency from line onset exceeded 3,000 ms (90% of responses in our pilot study were made within this window). To maximise the likelihood that time spent on task reduced alertness, participants who took a break that was longer than 5 min were also excluded from further data analysis. The length of this break was calculated as the difference between the last trial of the early block and the first trial of the late block.

The endpoint weightings exclusion criteria used by Mitchell et al. (2022) were applied, such that data were deemed invalid if the endpoint regression accounted for less than 70% of the variance in response position (r^2^ < .70), or if it produced an implausibly low value of EWS (<.50) or an implausibly large magnitude of EWB (absolute EWB > .50). Participants were excluded if they have invalid data in any condition, according to these criteria.

#### Endpoint weightings analysis

Within-subject factors were block (early, late) and presence of a tone (silent, tone). For each bisection, the horizontal position of the cursor position (P) was recorded relative to the screen centre. The endpoint weightings analysis was performed, per participant per condition, provided there were at least 32 valid trials available, otherwise the participant was excluded from the experiment. Endpoint weightings were extracted by fitting a linear regression with the left endpoint (L) and the right endpoint (R) of each line as predictors of P (Equation 1).

(1) $$P\;=\;\left({\mathrm{dP}}_{L}•\;L\right)\;+\;\left({\mathrm{dP}}_{R}•\;R\right)\;+\;k$$

In this equation, dP_L_ is the regression coefficient for the left endpoint, which defines the left endpoint weighting, while dP_R_ is the regression coefficient for the right endpoint, which defines the right endpoint weighting. The critical dependent measure is EWS, which is calculated by summing the left and right endpoint weightings (Equation 2).

(2) $$EWS=dP_{L}+dP_{R}$$

EWB was calculated as the right endpoint weighting minus the left endpoint weighting (Equation 3).

(3) $$EWB=dP_{L}-dP_{R}$$

Negative values of EWB indicate a stronger influence of the left endpoint, and positive values a stronger influence of the right endpoint. For exploratory purposes, we calculated the traditional measure of DBE as the deviation of the response (in mm) from the objective midpoint of the line, where positive values represent rightward errors, and negative values, leftward errors. The mean DBE was calculated per participant, per condition, as the unweighted mean DBE across the four-line types.

#### Hypotheses

To determine the effect of tonic and phasic arousal on both EWS (proposed measure of non-lateralised attention) and EWB (validated measure of lateralised attention), we tested four separate hypotheses.

##### Hypothesis 1: non-lateralised attention (EWS)

***Hypothesis 1.1***: If EWS is a valid index of non-lateralised attention, then it should be lower in the late block than in the early block, due to reduced tonic arousal and/or motivation. To test this hypothesis, we calculated the time-on-task effect per participant by subtracting EWS for silent trials in the early block from silent trials in the late block. The critical test of Hypothesis 1.1 was a one-sample *t*-test to test the prediction that the difference in EWS between the first and second blocks was significantly less than zero (*p* < .05). This would confirm the prediction that EWS is lower under conditions of lower tonic arousal.

***Hypothesis 1.2***: It is possible that the expected reduction in EWS between blocks is related to performance measures, rather than reduced motivation; therefore, we aimed to separately test the effect of increased arousal (phasic alerting on EWS). If EWS is a valid index of non-lateralised attention, then it will be higher under conditions of phasic alerting. To test this hypothesis, the mean EWS for silent and tone trials was calculated per participant by averaging EWS for these conditions across the two blocks, and the phasic alerting effect was calculated by subtracting EWS for silent trials from EWS for tone trials. The critical test of Hypothesis 1.2 was a one-sample *t*-test to test the prediction that the difference in EWS between silent and tone trials is significantly greater than zero (*p* < .05). This would confirm the prediction that EWS is higher under conditions of phasic alerting.

##### Hypothesis 2: lateralised attention (EWB)

***Hypothesis 2.1*:** If reduced tonic arousal increases rightward attentional bias, then EWB should increase (become more rightward) in the late block relative to the early block. To test this hypothesis, the same method for Hypothesis 1.1 was used for EWB.

***Hypothesis 2.2*:** If increased task arousal increases leftward bisection bias, then EWB should decrease under conditions of phasic alerting. To test this hypothesis, the same method for Hypothesis 1.1 was be used for EWB.

The statistical tests for Hypotheses 2.1 and 2.2 were the same as the critical tests for Hypothesis 1.1 and 1.2. In all cases, although we have a reason to expect an effect in one direction, we used a two-tailed test (with a significance criterion of *p* < .05), allowing for interpretation of effects in either direction.

It is worth noting that correction for multiple comparisons was not necessary here. This is because a conjunction of significant results was required for each measure (i.e., predicted effects confirmed for both tonic and phasic alerting) to test the hypothesis that non-lateralised attention influences that measure in the expected way. That is, to support out primary hypothesis that EWS is a valid measure of non-lateralised attention, we need to confirm that it is significantly reduced with time on task and significantly increased with phasic alerting. To support the idea that lateralised attention is yoked to non-lateralised alerting, we need to confirm that EWB is significantly increased with time on task and significantly reduced with phasic alerting. There are thus two separate hypotheses, for non-lateralised and lateralised attention, respectively, each of which depends on a conjunction of two sub-hypotheses, so multiple correction is inappropriate (Rubin, 2021).

### Recruitment, quality checks, and exclusions

The target sample size was 119, and we initially planned to collect data from up to 130 participants, to allow for approximately a 10% exclusion rate. This expected exclusion rate was much lower than our manipulation check, where most exclusions were due to participants failing the audio check. We updated our methods so that participants who failed the audio check did not proceed to the full experiment, so would not contribute to the total exclusion rate. However, in the first round of recruitment, 75 participants did not pass our other quality checks (see the *Quality checks* section), leaving only 65 valid datasets and <60% power to test our hypotheses. A protocol-amendment request to extend recruitment was approved on 18 November 2022, prior to any analysis beyond data-quality checks. This allowed us to recruit another 88 participants, up to a final total of 218 participants (not including 12 participants who did not complete the study), which exhausted our resources for the study.

A total of 230 participants took part in the experiment. Twelve of them failed the initial audio quality check and did not progress to the main experiment, meaning that 218 participants completed the experiment. Eighty-eight of these participants failed data-quality checks for the following reasons: 33 participants did not pass the screen calibration checks as the fit between their responses and the actual dot locations was less than r^2^ = .90; two participants took a break longer than 5 min between blocks; and 31 participants failed to identify the final audio check number correctly. After removing void trials and trials where response times were above 3,000 ms, 22 participants had too few trials remaining per condition (<32). Data from 130 participants, who passed initial data-quality checks, were analysed. A further 20 participants were removed at the analysis stage either because their endpoint regression accounted for less than 70% of the variance (r^2^ < .70), or they had an implausibly low EWS (<.50) or an implausibly high absolute EWB (>.50).

After all exclusions were made, our final sample size of *n* = 110 (55 women, 54 men, and 1 non-binary, mean age = 28.36 years, *SD* = 7.82, range = 19–59) was slightly below our original target sample size of 119. This provides 90% power to detect a minimum effect size of 0.31 (as opposed to 0.30).

### Results

All data and code for this manuscript are available through the Open Science Framework, which links to the projects GitHub repository (https://osf.io/sru9x/). The approved Stage 1 protocol can be found on the same OSF project page (for a direct link: https://osf.io/y2jc3).

**Figure 3.**
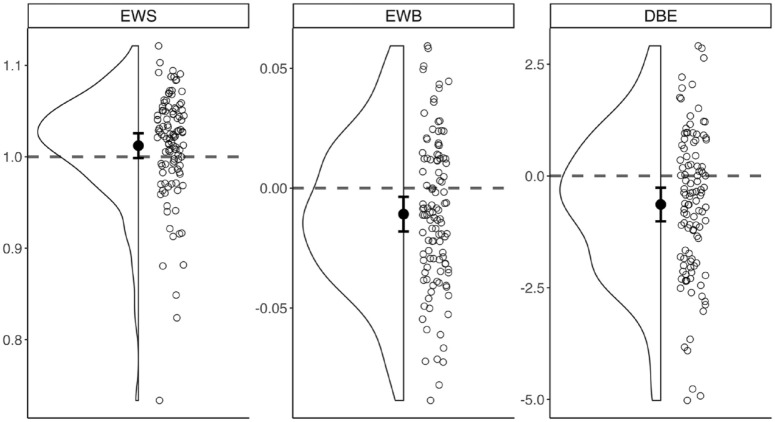
Distribution of EWS, EWB, and DBE. Unfilled dots show mean measurement in all participants, with the mean (black dot) and 95% confidence interval (error bars) of the sample for each measure. The scales for EWS and EWB are in the standardised units for these indices, and DBE is presented in millimetres.

For descriptive purposes, the distributions of EWS, EWB, and DBE are shown in Figure 3. As expected, the mean EWS was marginally above 1, and both mean EWB and mean DBE were slightly negative (consistent with pseudoneglect, e.g., Mitchell et al., 2022).

#### Hypothesis 1: the effect of arousal on non-lateralised attention

**Figure 4.**
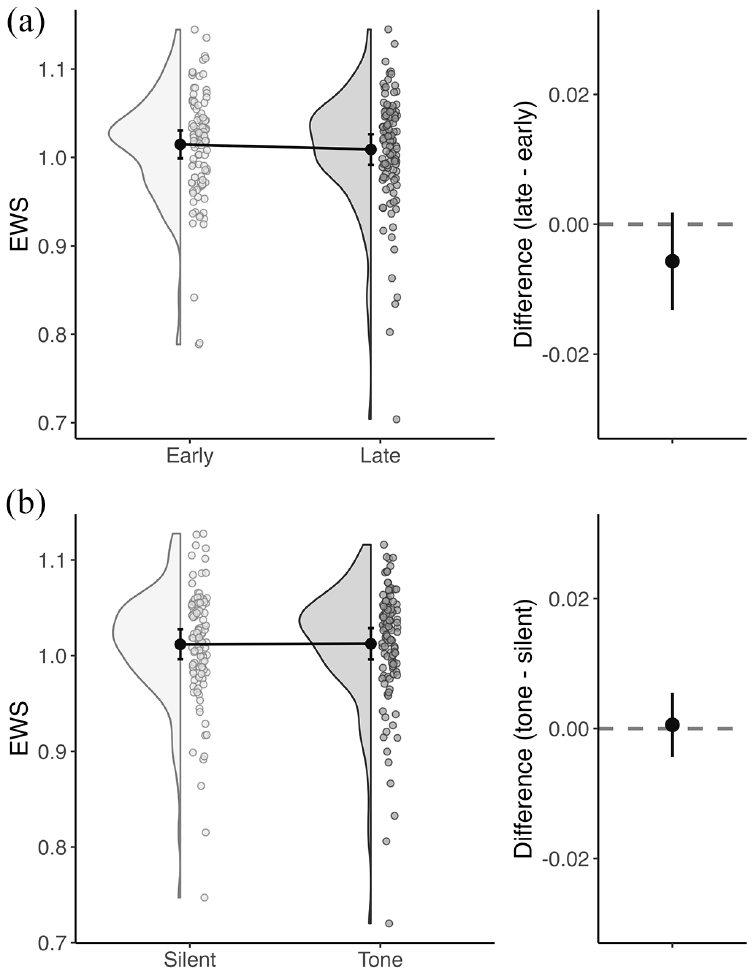
Non-lateralised attention. Summary of EWS distributions and differences between (a) early and late blocks and (b) silent and tone trials. Figures on the left show data from all participants as well as the group mean (black dot) for each condition, with 95% confidence intervals. Figures on the right show the mean difference in EWS between conditions, with 95% confidence intervals.

*Tonic arousal (Hypothesis 1.1)*: Only silent trials were used for this analysis. The difference between EWS in the late block compared to the early block (late − early, mean difference = −.01, *SD* = .04) was not significantly different from zero (t_109_ = −1.50, *p* = .14, *d* = −0.14). This means that EWS did not significantly reduce with time spent on task, and so EWS is not necessarily lower under conditions of reduced tonic arousal (Figure 4a).

*Phasic arousal (Hypothesis 1.2)*: The difference in EWS for tone trials compared to silent trials (tone − silent, mean difference = .00, *SD* = .03) was not significantly different from zero (t_109_ = .22, *p* = .82, *d* = .02), indicating that transient increases in phasic arousal induced by an alternating tone did not significantly increase EWS (Figure 4b).

#### Hypothesis 2: the effect of arousal on lateralised attention

**Figure 5.**
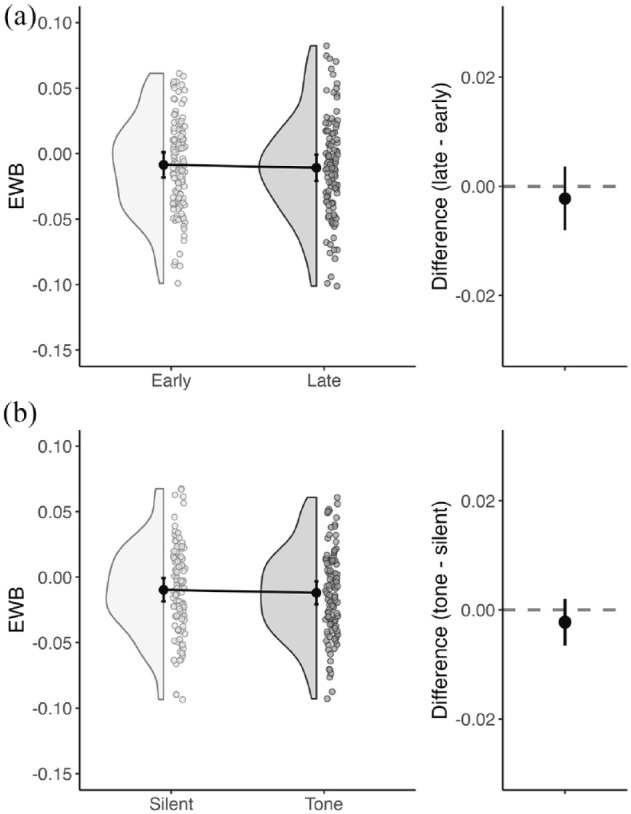
Lateralised attention. Summary of EWB distributions and differences between (a) early and late blocks and (b) silent and tone trials. Figures on the left show data from all participants as well as the group mean (black dot) for each condition, with 95% confidence intervals. Figures on the right show the mean difference in EWB between conditions, with 95% confidence intervals.

*Tonic arousal (Hypothesis 2.1)*: The difference in EWB between the early and late blocks (late − early, mean difference = .00, *SD* = .03), for silent trials only, was not significantly different from zero (t_109_ = −.76, *p* = .44, *d* = .07). Reduced tonic arousal in the late block did not induce a more rightward shift in lateralised attention (Figure 5a).

*Phasic arousal (Hypothesis 2.2)*: The difference in EWB between silent and tone trials (mean difference = −.00, *SD* = .02) did not significantly differ from zero (tone − silent, t_109_ = −1.03, *p* = .30, *d* = −.10), indicating the presence of an alerting tone, increasing phasic arousal, did not induce a leftward shift in lateralised attention (Figure 5b).

#### Exploratory analyses

##### The effect of tonic and phasic arousal on EWS and EWB

**Figure 6.**
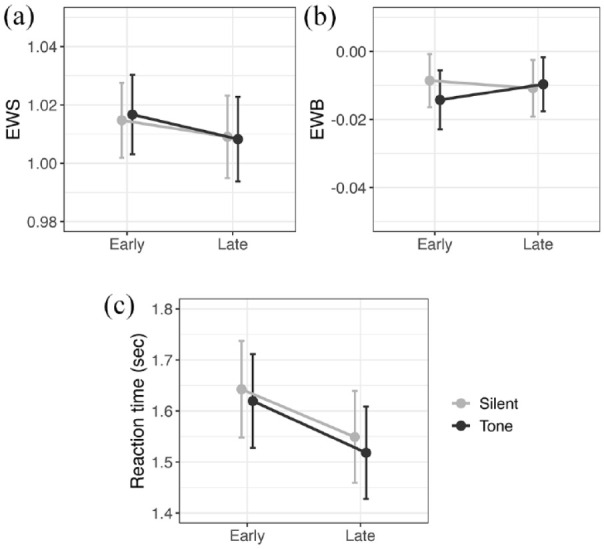
Interaction between tonic (early and late blocks) and phasic (silent and tone trials) arousal. Presented for EWS (a), EWB (b), and response time (c). Points show the mean of each condition for all participants. Error bars are 95% confidence intervals.

In addition to our registered analyses, we explored the effects of tonic and phasic arousal on EWS and EWB by conducting two within-subject two-way analyses of variance (ANOVAs) with the within-subject factors of block (early vs. late) and tone (silent vs. tone). This approach was not part of our initial registered analysis but has two benefits; it allows us to explore any possible interaction between manipulations of tonic and phasic arousal and to include all trials in the comparison between blocks, not just the silent trials, while accounting for any effect of the tone. Figure 6 shows the mean EWS (Figure 6a) and EWB (Figure 6b) across early and late blocks and in silent and tone trials.

Using this approach, EWS in the early block was found to be significantly higher than EWS in the late block (F_1,109_ = 5.96, *p* = .016, η^2^ = .05). That is, when both silent and tone trials are included in the analysis, there is evidence that EWS decreases with time on task, which follows our original predictions (mean difference between early and late blocks = −.01, *SD* = .03, Cohen’s d = −.23). No main effect of tone (F_1,109_ = .05, *p* = .82, η^2^ < .01) or interaction effects (F_1,109_ = .36, *p* = .55, η^2^ < .01) between block and tone were observed.

A similar analysis on EWB found no significant effects of block (F_1,109_ = 0.21, *p* = .65, η^2^ < .01) or tone (F_1,109_ = 1.07, *p* = .30, η^2^ = .01) or any interaction effects (F_1,109_ = 2.62, *p* = .11, η^2^ = .02).

##### The effect of time on task and tone on response time

Our registered analyses did not show the expected effects of our manipulations of arousal on EWS. One possible explanation for this could be that these manipulations were not effective in modulating participants’ arousal levels. To explore whether our chosen experimental manipulations increased or decreased arousal during the task, we explored the effect of time on task and tone trials on the response time between bisection stimulus and bisection response (Figure 6c).

Median response time was calculated per condition per participant, and a within-subject two-way ANOVA showed significant main effects of block and tone. Response time was significantly shorter in the late block than in the early block (F_1,109_ = 33.09, *p* < .001, η^2^ = .23), and in tone trials compared to silent trials (F_1,109_ = 6.59, *p* = .01, η^2^ = .06). No significant interaction between block and tone was observed (F_1,109_ = 0.25, *p* = .62, η^2^ < .01).

Therefore, although our manipulations of time on task and auditory tone were intended to have opposite effects to one another, leading to lower and higher alertness levels, respectively, the effect observed on response time was in the same direction. Faster responses were found with late blocks and toned trials. The possible implications of this pattern are considered in the Discussion section.

## Discussion

The endpoint weightings analysis method extracts a reliable and sensitive measure of lateralised attention bias during line bisection: EWB, the difference between the weightings assigned to the right and left line endpoints (McIntosh, 2018; McIntosh et al., 2005; Mitchell et al., 2022). This method also offers a second measure: EWS, the sum of the two endpoint weightings. In this study, we tested the hypothesis that EWS is a measure of generalised attention for the line-bisection task, by manipulating time spent on task to influence tonic arousal and using a short alerting tone to influence phasic arousal. Our registered analyses did not support our predictions: EWS did not significantly decrease between the early and late test blocks, and it did not significantly increase when the bisection stimulus was accompanied by an alerting tone. The null hypothesis that EWS is not influenced by changes to tonic or phasic arousal cannot be rejected, and our results suggest that EWS does not capture generalised attention to the task.

The lack of a clear effect of time on task on EWS contradicts previous studies on healthy individuals conducted both in the lab and online (McIntosh et al., 2019; Mitchell et al., 2022; Smaczny et al., 2024). For example, one recent study used a 50-minute vigilance task designed to decrease arousal (Smaczny et al., 2024). Participants took longer to respond to the vigilance task in the last 10 min, compared to the first, confirming a decrease in arousal. This reduction in arousal was coupled with a reduction in EWS, suggesting that EWS decreases with reduced tonic arousal. When we conducted an alternative exploratory analysis in the present study, which included all trials, a significant decrease in EWS with time on task was found, in accordance with our original predictions, and with the findings of Smaczny et al. (2024, also McIntosh et al., 2019; Mitchell et al., 2022). While we cannot drive strong conclusions from an exploratory outcome that differs from the main pre-registered outcome, this result is directionally consistent with prior evidence and implies that we should not rule out a prevailing reduction in EWS with time on task.

On the other hand, our results provided no significant support for an effect of alerting tone on EWS, in our registered or exploratory analysis. This does not support our previous lab-based result (Ahmad Khan, 2020). The online nature of the present study meant that we had less control over the auditory component of the task, than in a lab setting. Despite checks put in place to ensure that participants could hear the tone, uncontrolled factors such as external environment, the use of headphones, and sound location could potentially confound the results. Nonetheless, we did see a clear reduction in response time to tone trials compared to silent trials (Figure 6c), consistent with our preliminary manipulation check (see the “Preliminary manipulation check” section), which suggests that the tone was having the expected alerting effect. Another alternative explanation for this null finding is that EWS does not capture all types of attention allocated during line bisection and is, instead, selective for more long-term, endogenous attention (such as tonic arousal) than short bursts of exogenous attention typically elicited by an alerting tone. However, this suggestion is a speculative, post hoc adjustment to the proposed role of EWS.

Our exploratory analyses of response time not only revealed a clear alerting effect, with faster responses on tone trials, but also showed faster responses after more time spent on task (Figure 6c). This reduction of response time in later blocks of line bisection matches findings from our previous online study (Mitchell et al., 2022). At the time, we interpreted this reduction in response time as a withdrawal in attention from the task; i.e., a general disengagement reflecting less-careful responding. However, we acknowledge that it would be logically inconsistent to interpret faster response times as indicative of *both* an increased phasic arousal and reduced tonic arousal. We cannot rule out the possibility that the change in EWS over time observed in prior research (McIntosh et al., 2019; Mitchell et al., 2022; Smaczny et al., 2024) is not related to reduced attention to the task, but instead to improved task performance with practice, driving EWS closer to 1.00, indicating a closer approximation to ideal performance. Therefore, from the results of this present study, we cannot confidently conclude that the time-on-task manipulation produced the desired effect of a reduction in alertness. Overall, the primary takeaway from the present data is that, while there is tentative evidence that EWS decreases with reduced attention associated with time on task, there is no strong evidence to suggest that phasic arousal affects EWS.

With regard to lateralised attention, we found no evidence that changes to tonic or phasic arousal influence lateral biases of attention during line bisection. This contradicts previous work which suggests that manipulations of non-lateralised attention alter lateralised bias in healthy individuals (Benwell et al., 2013; Chandrakumar et al., 2019; Dufour et al., 2007; Manly et al., 2005). These previous claims have been based on a rightward shift of DBE under conditions of low arousal, for instance, after extended time on task, or reduced sleep. Here, we provide a high-powered online investigation, using manipulations of both tonic and phasic arousal, and find no clear effect of either manipulation on EWB. This is consistent with the result of a recent study, which also used EWB to measure lateral bias for line bisection and found no significant effect of reduced arousal (Smaczny et al., 2024). Our findings, along with those of Smaczny and colleagues, challenge the long-standing belief that non-lateralised attention affects pseudoneglect (Chandrakumar et al., 2019; Dodds et al., 2009; Manly et al., 2005; Schmitz et al., 2011).

Finally, we should consider the possible implications of having run this experiment online. We have shown previously that line bisection can be used online to measure pseudoneglect (Mitchell et al., 2022), which is confirmed by present results showing the expected leftward bias using EWB or DBE measures (Figure 3). Nonetheless, the relative lack of environmental control in an online setting means that our manipulations of tonic and phasic arousal are less well-controlled than in a laboratory. Given concerns about the quality of online data, we included strict quality checks to try to ensure that our manipulations were effective and that participants were engaging properly with the task. We anticipated a ~10% exclusion rate by these criteria but eventually excluded over 50% of participants. On the one hand, this very high exclusion rate inevitably lowers our overall confidence in the suitability of this experiment for an online format. On the other, the inclusion of rigorous quality checks should perhaps reassure us about the quality of the data on which our final inferential analyses were based. Despite such rigorous checks, it is still possible that the desired alerting effects were not elicited. For instance, while we can be confident that included participants did hear the tone, it might not have been at sufficient volume to produce phasic alerting effects during line bisection, which would affect both our measure of non-lateralised (EWS) and lateralised (EWB) attention. In addition to this, based on the exploratory response time analysis, it is unclear whether time-on-task manipulations resulted in improved or reduced levels of arousal. Therefore, while the present experiment did not support the validity of EWS as a measure of non-lateralised attention, this should not necessarily rule out a laboratory-based re-assessment of related questions.

To summarise, we manipulated both tonic and phasic arousal during an online line-bisection task, to test whether the EWS index of the endpoint weightings analysis is a valid measure of non-lateralised attention. The results of our main registered analyses revealed no significant effects of either manipulation, providing no support for the hypothesis that EWS is a valid measure of generalised attention. While our findings do not encourage the adoption of EWS as a measure of generalised attention, exploratory analyses revealed a possible effect of tonic alertness on EWS and call into question the effectiveness of online manipulations of alertness. Further lab-based manipulations of attention during line bisection may nonetheless be useful to increase our understanding of what aspects of behaviour EWS captures.

## Notes

1. The following pixel locations were presented, varying in horizontal (x) and vertical (y) axes, where [0,0] is the screen centre, negative x values are left, and negative y values are up: [0, −320], [0,0], [0, 320], [−480, 0], [−320, 0], [−192, 0], [192, 0], [320, 0], [480, 0]

2.
https://researcher-help.prolific.co/hc/en-gb/articles/360009223553-Using-attention-checks-as-a-measure-of-data-quality
